# Significance of Placental Mesenchymal Stem Cell in Placenta Development and Implications for Preeclampsia

**DOI:** 10.3389/fphar.2022.896531

**Published:** 2022-06-01

**Authors:** Yang Zhang, Yanqi Zhong, Li Zou, Xiaoxia Liu

**Affiliations:** Department of Obstetrics and Gynecology, Union Hospital, Tongji Medical College, Huazhong University of Science and Technology, Wuhan, China

**Keywords:** mesenchymal stem cell, placenta, preeclampsia, differentiation, paracrine

## Abstract

The well-developed placentation is fundamental for the reproductive pregnancy while the defective placental development is the pathogenetic basis of preeclampsia (PE), a dangerous complication of pregnancy comprising the leading causes of maternal and perinatal morbidity and mortality. Placenta-derived mesenchymal stem cells (PMSCs) are a group of multipotent stem cells that own a potent capacity of differentiating into constitutive cells of vessel walls. Additionally, with the paracrine secretion of various factors, PMSCs inextricably link and interact with other component cells in the placenta, collectively improving the placental vasculature, uterine spiral artery remolding, and uteroplacental interface immunoregulation. Recent studies have further indicated that preeclamptic PMSCs, closely implicated in the abnormal crosstalk between other ambient cells, disturb the homeostasis and development in the placenta. Nevertheless, PMSCs transplantation or PMSCs exosome therapies tend to improve the placental vascular network and trophoblastic functions in the PE model, suggesting PMSCs may be a novel and putative therapeutic strategy for PE. Herein, we provide an overview of the multifaceted contributions of PMSCs in early placental development. Thereinto, the intensive interactions between PMSCs and other component cells in the placenta were particularly highlighted and further extended to the implications in the pathogenesis and therapeutic strategies of PE.

## 1 Introduction

As the first organ to develop in mammals, the placentas are essential for anchoring the fetus, mediating the nutrient and waste exchange, and avoiding the rejection of the maternal immune system (Burton and Jauniaux, 2015). The placental functions are special and exact, which deeply relies on the integrity and coordination of the placental structures. The placenta is composed of numerous villous, which are functional units consisting of the outer epithelial trophoblast layer and stroma. Three main types of epithelial trophoblasts have been defined, including the syncytiotrophoblast (STB), cytotrophoblast (CTB), and extravillous trophoblast (EVT). The villous stroma contains endothelial cells (ECs), placenta-derived mesenchymal stem/stromal cells (PMSCs), Hofbauer cells amongst others (Burton and Fowden, 2015). Those different cell types all play their individual part in placentas but also inextricably link and interact with each other. However, the communication disturbance among those component cells in the placenta causes defective placentation and placental dysfunction, which is one of the main causes of multiple obstetric complications such as preeclampsia (PE). Thereinto, PMSCs, a group of fibroblast-like cells with multipotential differentiation and paracrine mechanism (Lankford et al., 2017), are closely involved in placentation. PMSCs may differentiate into the constitutive cells of vessel walls to initiate early placental vasculogenesis (Boss et al., 2018). Besides, PMSCs interact with other component cells in the placenta *via* their active paracrine, thus improving the placental angiogenesis, augmenting the uterine spiral artery remolding, and modulating the uteroplacental immune status (Magatti et al., 2019; Wu et al., 2020; Liu et al., 2021). Preeclamptic PMSCs have been reported to be dysfunctional and senescent with detrimental paracrine, thus playing a vital role in the development and severity of PE (Wang et al., 2012; Rolfo et al., 2013; Ji et al., 2017; Nuzzo et al., 2017). However, PMSCs transplantation or PMSCs exosome therapies tend to improve the PE symptoms and pregnancy outcomes in the PE model, suggesting PMSCs may be a potential therapeutic strategy for PE. Nevertheless, PMSCs are still a rather poorly understood cell type in the physiological and pathological functions of the placenta. In this review, we provide an overview of the multifaceted contributions of PMSCs in early placental development. We highlight the intensive interactions between PMSCs and other component cells in the placenta, and extend the significance of these links during placentation as well as implications for the pathogenesis and therapeutic strategies of PE.

## 2 Pathogenesis of Preeclampsia

PE, one of the leading causes of maternal and perinatal morbidity and mortality (Gestational Hypertension and Preeclampsia, 2020), is new-onset hypertension typically after 20 weeks of gestation, accompanied by multisystem signs or symptoms, including proteinuria, elevated liver enzymes, renal insufficiency, thrombocytopenia, and even maternal and fetal death (Zhang et al., 2020). PE not only elevates the risk of cardiovascular and metabolic diseases in mothers in subsequent years (Wu et al., 2017; Abbasi, 2018) but also has long-term effects on the offspring concerning cardiometabolic health (Davis et al., 2015; Rice et al., 2018) and neurodevelopment (Sun et al., 2020). The precise pathogenesis of PE remains vague and complicated while the two-stage theory of preeclamptic pathogenesis proposed by Redman has been widely acknowledged; that is, the first stage mainly comprises impaired placentation, contributing to the excessive secretion of proinflammatory and antiangiogenic factors, such as soluble fms-like tyrosine kinase-1 (sFlt-1) and soluble endoglin (sENG), into the maternal circulation to cause the multiple clinical manifestations in the second stage (Redman and Sargent, 2009). Thereinto, the two-phase theory highlights the deficient placental development as the fundamental part of the PE pathogenesis (Fisher, 2015), which may be closely linked with the dysfunction of component cells in the placenta (Burton et al., 2019). Besides, a multitude of placental cell types may not only be independent and separate factors but also keep intricate communication with each other. Owing to the multidirectional differentiation and paracrine actions, PMSCs might act as the core in cellular interactions in the placenta. PMSCs have been reported to be critical for successful pregnancies and defective in PE (Rolfo et al., 2013). Nonetheless, little is understood about the role of PMSCs in placental development or dysfunction.

## 3 What are PMSCs?

“PMSCs” is usually used as a general term including all the mesenchymal stem cells (MSCs) isolated from different sites of the placenta, such as the chorionic membrane (CMMSCs) (Jaramillo-Ferrada et al., 2012), the chorionic villi (CVMSCs) (Makhoul et al., 2013), chorionic cotyledons or intervillous space (CIVMSCs) (Chen et al., 2016; Mathew et al., 2017), chorionic plate (CPMSCs) (Kim et al., 2011), the chorionic trophoblastic cells (Knöfler et al., 2019), the amniotic membrane (AMMSCs) (Tamagawa et al., 2004), the amniotic fluid (AFMSCs) (Moraghebi et al., 2017), the umbilical cord (UCMSCs) (Wang et al., 2020), and the decidua basalis (DBMSCs) (Abumaree et al., 2017). Though derived from different parts in the placenta, all these cells conform to the definition of MSCs according to the International Society for Cellular Therapy (ISCT) (Dominici et al., 2006). PMSCs can be induced to differentiate into three lineages: chondrocytes, adipocytes, and osteocytes *in vitro*. PMSCs can express CD73, CD90, and CD105 markers but not markers like CD45, CD14, CD19, CD34, and HLA-DR (Abumaree et al., 2013b). Nonetheless, only a few comparative studies of PMSCs from different sites in the placenta have been performed to date, leading to a limited understanding of what specific cells have been used or what kind of better. Herein, we have tried to refer to specific types wherever available while the general term “PMSCs” has still been inevitably used in most cases.

Bone marrow-derived mesenchymal stem cells (BMMSCs) have long been considered the gold standard in research activities related to MSCs while obtaining bone marrow has been a challenge, accompanied by the inevitable ethical issue, low MSCs production, and several risks (Mathew et al., 2020). Besides, affected by the donor’s age, the capacity for proliferation and differentiation is always limited in the adult MSCs. Fetal stem cells have been proven to be more primitive and more potent than their adult counterparts (Abdulrazzak et al., 2010). Placenta, a transient feto-maternal organ, is discarded after delivery without invasive procedures, making it easily more available and ethically more favorable with a rich supply of MSCs(Parolini et al., 2008). Compared to the BMMSCs counterparts, PMSCs have now gained increasing attention for their abundance, ease of availability, low immunogenicity (Le Blanc and Ringdén, 2005), effective immunomodulatory ability (Lee et al., 2012), long-term growth ability (Barlow et al., 2008), slow aging rate (Batsali et al., 2017), and potent expansion ability (Talwadekar et al., 2015). Intriguingly, PMSCs are less prone to osteogenic, adipogenic, and myogenic differentiation than BMMSCs but reveal strong endothelial conversion properties (Castrechini et al., 2010; Pilz et al., 2011; Meraviglia et al., 2012; Hart et al., 2017) both *in vitro* and *in vivo*, which is due to the microenvironment and cellular niches affecting their fate (Lin, 2002). On the one hand, the placenta is a highly vascular organ and PMSCs, residing in the placental vascular niche (Castrechini et al., 2010), may participate in forming the original vessels in the placentas. On the other hand, PMSCs secrete a plethora of bioactive molecules, thus promoting the formation of placental development and angiogenesis (Demir et al., 1989; James et al., 2014; Aplin et al., 2015; Boss et al., 2018; Mathew et al., 2020). Hence, we are particularly interested in the role of PMSCs in placentation and placental functions.

## 4 How do PMSCs Play a Role in Placentation, Placental Function and Dysfunction?

### 4.1 PMSCs Participate in the Placentation *via* Differentiating Into Constitutive Cells of Vessel Walls

Placental vasculature development is essential for successful pregnancy while impairments of the placental vessel are implicated in gestational complications such as PE. Circulating endothelial progenitor cells (EPCs) originating from bone marrow have long been considered the main executor of vasculogenesis, mobilizing to create or incorporate into vessel walls (Liu et al., 2015; Liu et al., 2017). Circulating maternal/fetal EPCs were previously proposed as the origin of the placental vasculature, provided that both the fetal-placental and uteroplacental circulation are well-connected. On the one hand, beginning at 6–8 weeks and completing by 19–20 weeks of gestation (Harris, 2010), human trophoblasts invade and remold the uterine spiral arteries, resulting in the uteroplacental circulation in the intervillous space where the maternal blood is free to interact with the placental villous (Degner et al., 2017). On the other hand, vasculogenesis can be seen in the yolk sac at around day 18–20 of gestation and umbilical cord vessels do not connect the embryo to the placenta until almost day 32 of gestation, representing the establishment of the fetal-placental circulation (Dempsey, 1972; Boss et al., 2018). However, the formation of primitive endothelial cell cords in early placental development could be found as early as day 15 of gestation, indicating that placental endothelial cells may not the origin from EPCs at that time. MSCs from multiple sources show the potential of endothelial differentiation (Wang et al., 2018), among which PMSCs reveal strong endothelial conversion properties both *in vivo* and *in vitro* (Castrechini et al., 2010; Pilz et al., 2011; Meraviglia et al., 2012; Hart et al., 2017). Treated by the endothelial differentiation system supplemented with single or multiple cytokines, PMSCs can differentiate into cells expressing endothelial markers such as CD31, CD34, CD144, von Willebrand factor (vWF), vascular endothelial growth factor receptor-1 (VEGFR1), VEFGR2, and endothelial nitric oxide synthase (Chen et al., 2009; Benavides et al., 2012; Du W. J. et al., 2016). Endothelial differentiated PMSCs have enhanced the capacity to form tube-like structures on Matrigel gel (Liang L. et al., 2017) and actively engaged in the placental vessel walls *in vivo* (Liu et al., 2021). Given the superior multipotential differentiation of the primitive villus mesenchyme, PMSCs have been gradually accepted to be the origin of early placental endothelial cells (Dempsey, 1972; Boss et al., 2018). Therefore, PMSCs are mostly likely to initiate forming the original vessels in the early placental development. Nonetheless, future studies are still necessary to shed further light on how endothelial cell lineage differentiation events occur very early in gestation.

Vascular smooth muscle cells (VSMCs), residing in the perivascular niche of mesenchymal villi, regulate vessel stability and blood flow in the placenta (Hamilton et al., 2010). It is quite hard to define when the VSMCs initially appear in the developing placenta since cells expressing contractile/mature VSMCs markers (α- and γ-smooth muscle actin) have not been described in the early placenta until 6 weeks in gestation, suggesting that VSMCs may derive either from the mesenchymal core or embryo *via* umbilical circulation (Boss et al., 2018). Induced by transforming growth factor-β1 (TGF-β1), platelet-derived growth factor (PDGF)-BB, or the matrix coated with collagen I, collagen IV, and laminin, PMSCs also possess the potential of differentiating into VSMCs with the expression of myofibroblast/smooth muscle markers *in vivo* and *in vitro* (Chen C. Y. et al., 2015; Xie et al., 2016; Boss et al., 2020). Functionally, MSCs-derived VSMCs displayed contracting capacity and supported vascular structure formation in the Matrigel plug assay *in vitro* and also gave rise to the smooth muscle layer of vascular grafts *in vivo* (Gu et al., 2018). Reportedly, in response to vascular injuries, it is the MSCs’ differentiation into VSMCs rather than the proliferation of VSMCs themselves that contributes to the vascular remodeling (Tang et al., 2012). Therefore, PMSCs may provide a source of VSMCs in the placenta throughout gestation while future work is needed to more clearly and fully understand the relationship between the PMSCs and VSMCs in the placenta.

Unlike the VSMCs altering blood flow in larger vessels, pericytes are cells present at intervals along the walls of arterioles and capillaries (Barreto et al., 2019). Pericytes modulate vascular stability, permeability, and blood flow control in the placenta (Pallone et al., 2003). Prior to the establishment of the umbilical circulation, cells akin to pericytes can be found to be contacted with PMSCs in perivascular niches (Demir et al., 1989). These cells, are polygonal, arranged in a weblike shape, and surrounding the forming vessel wall to support the developing placental vessels network (Demir et al., 1989). It is quite difficult to distinguish pericytes and MSCs to some extent since they both reside in the perivascular niche, express many of the same markers, and demonstrate the ability of multiple lineage differentiation (Castrechini et al., 2010; Chang et al., 2014). Pericytes are very likely to be differentiated from MSCs(Ding et al., 2004) while others found that pericytes can also act as a source of MSCs and differentiate into cells of mesenchymal origin (Feng et al., 2011). Hence, PMSCs and pericytes may be inseparable and interconvert with each other in the vascular niches of placental chorionic villi.

In sum, all those abovementioned strongly suggest that PMSCs may differentiate into the constitutive cells of vessel walls (ECs, VSMCs, or pericyte) in the early placenta, at least prime for differentiation towards these lineages, thus initiating the vasculogenesis or stabilizing nascent vascularization by functioning as perivascular precursor cells.

### 4.2 PMSCs Modulate Placental Development and Functions *via* Their Paracrine Mechanism

The paracrine effect of MSCs is the most comprehensive and enduring mode of action. On the one hand, MSCs are known to secrete a plethora of bioactive molecules, such as immunomodulation factors, angiogenic factors, antiapoptotic factors, antioxidative factors, and chemokines (Liang et al., 2014). Depending on the tissues from which MSCs are isolated, MSCs may secrete various tissue-specific factors. For instance, PMSCs may produce less vascular endothelial growth factor (VEGF), tumor necrosis factor-α (TNF-α), interleukin (IL)-6 receptor, and IL-13, compared to the BMMSCs counterparts, while mainly produced factors such as hepatocyte growth factor (HGF), basic fibroblast growth factor (bFGF), IL-6, IL-8, IL-1α, IL-1β, and cyclooxygenase-2 (Cox-2) (Du W. J. et al., 2016; Saleh et al., 2020). On the other hand, current research is now shifting gears and looking at more specific components of the paracrine content from PMSCs, most notably the exosomes. These are extracellular vesicles (EVs), much smaller than the microvesicles (>200 nm), ranging from 50 to 200 nm in diameter, processed through several endocytic steps before being released. EVs contain various molecules like lipids, proteins, DNA, mRNAs, long non-coding RNAs (LncRNAs), and micro RNAs (miRNAs), which are delivered to target cells to be involved in several functions (Yu et al., 2014; Phinney and Pittenger, 2017). The paracrine capacity of PMSCs has long been proposed as the principal mechanism in cellular crosstalk with other cell types in the placenta, contributing to placental development and functions in normal pregnancy.

Reportedly, the quantification and function of PMSCs are affected by gestational age. Concretely, first trimester-derived PMSCs and term PMSCs counterparts both displayed analogous mesenchymal, perivascular, immunological immunophenotypes, and mesenchymal lineage differentiation potential (Hong et al., 2013). However, the concentration, migration, and proliferative abilities of PMSCs decreased as gestation proceeded to term (Ma et al., 2012; Iwatani et al., 2017b; Mohammadi et al., 2018). Besides, first trimester-derived PMSCs were more efficient in their *in-vitro* differentiation toward selective mesenchymal cell types, as well as toward neuronal-like and hepatocyte-like lineages (Hong et al., 2013; Iwatani et al., 2017a). Therefore, a restriction in multipotentiality of PMSCs is imposed *via* gestational age, thereinto, first trimester-derived PMSCs show more plasticity than term PMSCs counterparts concerning fate acquisition. Even though the physiological senescence exists in the DBMSCs as pregnancy advances to term (Khanabdali et al., 2021), preeclamptic PMSCs exhibit abnormal proliferation, inhibited migration, and accelerated senescence (Wang et al., 2012; Rolfo et al., 2013; Ji et al., 2017; Nuzzo et al., 2017; Romberg et al., 2022). Senescent PMSCs in PE are further characterized by abnormal paracrine actions. Hwang et al. (2010) have reported that preeclamptic DBMSCs had significantly reduced levels of soluble intracellular adhesion molecule-1 (sICAM-1) and stromal cell-derived factor-1 (SDF-1, the ligand for chemokine receptor CXCR4. Rolfo et al. (2013) have found that preeclamptic PMSCs had increased release of proinflammatory cytokines compared to normal PMSCs, probably associated with some of the immunological alterations in PE. Besides, research on EVs derived from preeclamptic PMSCs has also enumerated several exosomal miRNAs and LncRNAs with aberrant expression, resulting in the dysfunction of other cell types in the placenta (Liu et al., 2012; Wang et al., 2012; Ji et al., 2017; Li et al., 2017; Nuzzo et al., 2017; Qu et al., 2018; Cui et al., 2020; Wang et al., 2020; Yang et al., 2021). Hence, the paracrine in preeclamptic PMSCs may disturb the interaction between PMSCs and other placental cells. This abnormal interaction may be detrimental to the placentation and placental function, thus exacerbating the development and severity of PE.

#### 4.2.1 Interaction Between PMSCs and EPCs

As the placenta is a highly vascular organ capable of producing hematopoietic cells (Gao et al., 2018), it is possible that the placenta is a primary or supplementary source of EPCs, or at least retains EPCs on passage through the placenta itself. Fetal EPCs and maternal circulatory EPCs may both play an integral role in placental endothelium regeneration, vessel repair, and maintenance (Hubel et al., 2011). However, a lower quantification (King et al., 2013) and reduced vasculogenic capacities (Liu et al., 2015) of EPCs were found in PE patients (Sugawara et al., 2005; Liu et al., 2016).

*In vitro* researches are usually the coculture of MSCs and EPCs (Shafiee and Khosrotehrani, 2017), in which Hou et al. (2017) have reported that MSCs improved the survival and capillary formation of EPCs via secreting insulin-like growth factor-1 (IGF-1) to activate PI3K/Akt signaling pathway while Liang et al. have found the underlying mechanisms may owing to PDGF and NOTCH signaling (Liang T. et al., 2017). Ge et al. (2018) have further indicated that MSCs produce VEGF to promote EPCs differentiating into ECs, which lay the basis for the co-transplantation of MSCs and EPCs *in vivo.* Compared to the single EPCs or PMSCs transplantation, co-transplantation significantly elevated the engraftment, maintained endothelial phenotype, and generated functional vasculature (Gao et al., 2019). The possible reasons may be described as follows. Firstly, MSCs and EPCs tend to be attached to each other, partly mediated through the E-cadherin (E-cad)/beta-catenin signaling pathway (Xia et al., 2016). Fibronectin in EPCs may also activate the integrin α5β1 of MSCs to further mediate cell-cell cohesion and crosstalk (Zhang H. W. et al., 2015). Secondly, IFN-γ induced HLA-DR expression on EPCs and MSCs respectively, but both cell types had significantly less HLA-DR in cocultures (Souidi et al., 2017). Thus, MSCs may not only enhance the vasculogeneic and proangiogenic activities of EPCs but also act as guardians for EPCs by protecting the EPCs in immunocompetent hosts. Thirdly, EPCs can also serve as paracrine mediators and regulate the regenerative potential of MSCs via PDGF-BB/PDGFR-β signaling (Lin et al., 2014). Hence, in the context of placental development, PMSCs and EPCs are very likely to interact with each other to jointly obtain a synergistic effect in terms of angiogenesis (Sun et al., 2016), thus generating a favorable environment for the placental vasculature formation.

#### 4.2.2 Interaction Between PMSCs and ECs

Localized perivascularly in placental tissues, PMSCs inextricably interacted with the components of vessel walls *via* paracrine mechanisms. Conditioned medium of PMSCs augmented ECs proliferation, migration, and tube formation *in vitro* (Wu et al., 2020), which may owe to the abundant angiogenic factors at bioactive levels in PMSCs culture, including but not limited to VEGF, angiogenin, bFGF, IGF-1, insulin-like growth factor binding protein 2 (IGFBP2), IGFBP3 and IGFBP6(Kong et al., 2013; König et al., 2015; Komaki et al., 2017; Ma et al., 2021). Additionally, VCAM-1 may serve as a marker of PMSC subpopulation with superior angiogenic potential since VCAM-1^+^ CVMSCs population showed remarkable vasculo-angiogenic abilities both *in vitro* and *in vivo* with upregulated expression of angiogenic genes and increased secretion of proangiogenic cytokines (Du W. et al., 2016). Transplantation of PMSCs incorporated into diabetic Goto-Kakizaki rats’ vasculature with improved angiogenesis, which can be attributed to the direct *de novo* differentiation and proangiogenic paracrine actions (Kong et al., 2013). The exosomes of PMSCs can be incorporated into ECs *in vitro*, stimulating endothelial migration, tube formation, and angiogenesis-related gene expression (Zhang B. et al., 2015). Besides, the PMSCs exosome infusion in an *in-vivo* murine auricle ischemic injury model also enhanced angiogenesis (Komaki et al., 2017). Hence, PMSCs, in normal conditions, have a protective effect on endothelial functions. However, the real crosstalk between PMSCs and ECs in the PE placenta may deviate from the normal. Compared with healthy donors, miR-136 and miR-16 are both highly expressed in DBMSCs from PE, and the conditioned supernatants of which also inhibit the capillary formation of HUVECs (Wang et al., 2012; Ji et al., 2017). Also, miR-494 is highly expressed to arrest G1/S transition in the preeclamptic DBMSCs by targeting CDK6 and CCND1, supernatant of which also impairs capillary formation by suppressing VEGF (Chen S. et al., 2015). Therefore, preeclamptic PMSCs may induce endothelial dysfunction to impair the placental vasculature.

Vasculogenesis and angiogenesis are two consecutive processes during the development of the placental vascular network. The first step, vasculogenesis, is the formation of the first blood vessels achieved by the differentiation of pluripotent mesenchymal cells, which partly rely on the differentiation of PMSCs in the early placenta development. Angiogenesis is the second step characterized by the development of new vessels from already existing vessels, in which PMSCs closely communicate with EPCs and ECs to directly improve the placental vasculature formation by active paracrine (Demir et al., 2007). There is no doubt that PMSCs play a fundamental role in the placental vasculature formation of the normal pregnancy. Even though no comparison has been reported yet on the potency of endothelial differentiation between the normal and PE PMSCs, the preeclamptic PMSCs restrain the placental vessel formation at least partly via their detrimental paracrine.

#### 4.2.3 Interaction Between PMSCs and Trophoblasts

In the early placental development, proliferative CTB makes up the primary villi, and villous CTB fuses into STB to mediate the transport of oxygen and nutrients, production of gestational hormones, and clearance of fetal waste (Aplin, 2010). EVTs are migratory and invasive trophoblasts, invading and remolding the uterine spiral arteries, veins, decidual lymphatics, and glands, which are temporally and spatially affected by the uteroplacental environment. Trophoblastic dysfunction and defective spiral artery remodeling have been widely reported in PE (Zhang et al., 2021a).

Placental development is exponential in the first trimester and involves coordinated events between the trophoblasts and villous mesenchyme (Ding et al., 2021). The UCMSCs’ supernatant or coculture with UCMSCs both remarkably enhanced the trophoblastic proliferation and upregulated their invasive abilities (Chen, 2014; Huang et al., 2016). PMSCs increase the mitochondrial function in trophoblasts via enhancing adenosine triphosphate (ATP) synthesis and inducing mitochondrial mitophagy, the underlying mechanism may be due to the balance between protein tyrosine phosphatase (PTEN)-induced putative kinase 1 (PINK1) and parkin RBR E3 ubiquitin-protein ligase (PARKIN) expression (Seok et al., 2020; Seok et al., 2021). Also, PMSCs secrete HGF and increase trophoblastic cyclic adenosine monophosphate (cAMP) production, further modulating the trophoblastic adhesion and migration *via* signaling to Rap1 and integrin-β1 (Chen et al., 2013). Thus, PMSCs may maintain the metabolism homeostasis in trophoblasts to improve their biological function. Exosomal miR-139-5p from UCMSCs also accelerated trophoblastic invasion and migration by the motivation of ERK/MMP2 pathway via PTEN downregulation (Liu et al., 2020). In sum, in normal conditions, PMSCs conduce to the trophoblastic function. However, the conditioned medium of preeclamptic PMSCs has been found to impair the migrative and invasive abilities of trophoblasts (Wang et al., 2012; Ji et al., 2017). The abnormal Cyclin D1-p16INK4A/p18INK4C expression in placental explants conditioned by PE-PDMSCs’ medium suggests a negative contribution of preeclamptic PMSCs, which altered trophoblast cell cycle regulation (Nuzzo et al., 2017). Elevated Notch2, TIM3, and mTORC1 levels, and down-regulated miR-18b expression have been found in the EVs of Preeclamptic PMSCs compared with the normal PMSCs, which were delivered to trophoblasts to inhibit trophoblastic proliferation and migration (Yang et al., 2021). UCMSCs-derived exosome-mediated transfer of miR-133b enhanced trophoblast cell proliferation, migration, and invasion by restricting serum and glucocorticoid-regulated protein kinase 1 (SGK1) (Wang et al., 2020), and miR-101-containing EVs derived from UCMSCs bind to BRD4 and enhance proliferation and migration of trophoblasts (Cui et al., 2020). However, miR-133b and miR-101 was both reduced in preeclamptic UCMSCs exosome (Cui et al., 2020; Yang et al., 2021). Hence, cellular communication between PMSCs and trophoblasts is beneficial to early placental development in the normal pregnancy, while the pernicious paracrine in the preeclamptic PMSCs may cause trophoblastic dysfunction and jeopardize the placental homeostasis.

#### 4.2.4 Interaction Between PMSCs and Immune Cells

During productive pregnancy, it is vital to successfully establish the coexistence between the semi-allogenic fetus and the mother, which requires a dynamic immune system to guarantee immune tolerance. However, the mother failing to develop immune tolerance to the paternal genes of the embryo is more likely to have defective placentation in PE (Saito et al., 2007). The precise mechanism of fetal-maternal tolerance remains unclear. The immunomodulatory properties of PMSCs have now been valued, as well as the concerted action with the major immune cell subsets present in the uterus during the placental development, such as natural killer (NK) cells, macrophages, and T cells.

##### 4.2.4.1 Interaction Between PMSCs and NK Cells

Decidual natural killer (dNK) cells account for up to 70% of the lymphocytes present in the decidua in the early placental development (Kwan et al., 2014), which play a fundamental role during pregnancy, such as the induction of tolerance at the utero-fetal interface, promotion of spiral artery remodeling, and secretion of angiogenic factors to induce the neo-angiogenesis in the placental development (Karimi and Arck, 2010; Montaldo et al., 2014). Generally, peripheral blood NK (pbNK) cells express low levels of CD56 and are positive for CD16 (CD56^dim^CD16^+^), which exhibit high cytotoxic activity to kill non-self and transformed cells lacking human leukocyte antigen (HLA)-I molecules and expressing ligands for activating NK receptors (Vivier et al., 2018). However, the majority of dNK cells are CD56^bright^CD16^−^. Compared to the pbNK counterparts, dNK cells appear to be poorly cytotoxic with the expression of inhibitory receptors, including the leukocyte immunoglobulin-like receptor subfamily B member 1 (LILRB1), recognizing HLA-G, and KIR, in particular KIR2DL4, that binds to HLA-C molecules (Vacca et al., 2008). Therefore, though trophoblast cells express both HLA-G and HLA-C, dNK cells tend to show preferential tolerance towards trophoblasts. Nonetheless, the lack of uterine NK cells or alterations in dNK cell numbers and activation status all can lead to pathological changes in the placenta and subsequent gestational complications such as PE (Jabrane-Ferrat, 2019).

During the development of the placenta, PMSCs are in strict proximity with dNK cells to play a role in pregnancy maintenance. Firstly, PMSCs may facilitate the generation of dNK cells. DBMSCs can maintain the differentiation of CD34^+^ hematopoietic precursors derived from decidua towards functional CD56^bright^CD16^−^KIR^+/−^ NK cell (Vacca et al., 2011). Also, DBMSCs produced TGF-β to convert CD16^+^pbNK cells into CD16^−^dNK-like phenotype cells by triggering the expression of CD9 and KIR (Keskin et al., 2007). In the presence of cytokines essential for NK cells expansion (IL-2, IL-3, IL-15, and FLT3 ligand), AMMSCs can efficiently augment the differentiation and expansion of NK cell progenitors originated from umbilical cord blood (Ahmadi et al., 2015). Secondly, PMSCs may improve the lymphocyte viability and protect dNK cells from apoptosis via releasing plenty of soluble factors including prolactin, IGF-1, VEGF, and IL-11 (Magatti et al., 2019). Thirdly, PMSCs may modulate the cytotoxicity of dNK cells in the placenta. DBMSCs can effectively inhibit the expression of natural cytotoxic receptors, production of levels of perforin and granzymes, and secretion of inflammatory cytokines in dNK cells, the underlying mechanism may owe to DBMSCs constitutively producing indoleamine 2,3-dioxygenase (IDO), prostaglandin E2 (PGE2), and MCP-1 (Chen et al., 2011; Xu et al., 2012; Croxatto et al., 2014). Similarly, both AMMSCs and UCMSCs suppress the cytotoxicity and activation status of NK cells, thus reducing the production of IFN-γ, TNF-α, and perforin (Noone et al., 2013; Ribeiro et al., 2013; Chatterjee et al., 2014a; Chatterjee et al., 2014b; Li et al., 2015). Hence, the suppressive actions of PMSC and their secreted factors on dNK cell proliferation, phenotype, and cytotoxicity may illustrate the crucial role of PMSCs in placental development.

##### 4.2.4.2 Interaction Between PMSCs and Macrophages

Macrophages account for 20%–25% of the total leukocyte population in the first trimester and sustain the presence throughout pregnancy (Williams et al., 2009). As one of the vital components of the immunological milieu of pregnancy, macrophages contribute to the fetal-maternal tolerance, assist the embryo implantation, and modulate the spiral artery transformation (Erlebacher, 2013; Ning et al., 2016). With the variation of surroundings, macrophages tend to gain distinct cellular phenotypes, the functional spectrum of which ranges from proinflammatory M1 to anti-inflammatory tissue healing M2 (Ferrante and Leibovich, 2012). M1 phenotype is the predominant decidual macrophage during the peri-implantation period. After the trophoblastic invasion, a mixed M1 and M2 polarization pattern in decidual macrophages remains until mid-pregnancy. When placental development is completed, decidual macrophages are mainly polarized toward an M2 phenotype for the immune tolerance towards the fetus and successful parturition (Jaiswal et al., 2012). Thus, decidual macrophages exhibit a prevailing type of immunosuppressive phenotype and M2 polarization, which is instrumental for immunoregulation during early placental development. Similarly, Hofbauer cells, the macrophages in the chorionic villi, also possess an M2 phenotype to regulate fetoplacental angiogenesis and chorionic villus growth (Loegl et al., 2016). Reportedly, the altered macrophage numbers, aberrant macrophage activation, or the imbalanced M1 and M2 polarization pattern all can induce multiple pregnancy-related complications such as PE (Nagamatsu and Schust, 2010; Young et al., 2015).

On the one hand, PMSCs and their secreted factors block monocytes differentiating towards M1 macrophage but induce the monocytic differentiation into macrophages enriched with anti-inflammatory M2-like phenotype (CD14^high^CD163^+^CD209^+^). Reportedly, exposed to CVMSCs or AMMSCs *in vitro*, monocytes showed M2-like features similar to the macrophages in placental tissues, such as the expression of M2 markers (CD14, CD23, CD163, and CD209), an elevated expression of co-inhibitory molecules (B7-H4, PD-L1, and PD-L2), a reduced expression of co-stimulatory molecules (CD40, CD80, and CD86), the higher phagocytic activity, the increased production of immune-regulatory factor, as well as the decreased secretion of proinflammatory cytokines (Abumaree et al., 2013a; Pianta et al., 2016; Magatti et al., 2017). On the other hand, PMSCs promote a phenotypical and functional switch from M1 to M2 macrophages (Lindau et al., 2018; Pampalone et al., 2021). Intriguingly, murine macrophages also go through the M2 polarization when co-cultured with human PMSCs (Li et al., 2013; Onishi et al., 2015; Huang et al., 2021a). Thereinto, constitutively-produced bioactive factors of PMSCs may play a key role in their immunomodulatory activity, which may owe to the HGF, proteinoids (PGD2, PGF2a, and PGE2), IL-6, M-CSF, IL-10, and TSG-6 (Huang et al., 2021a). Recently, chie et al. have found that PMSC-derived Slit2 might suppress migration and enhance adhesion *via* modulating the activity and motility of inflammatory macrophages in the placenta (Chen et al., 2021). This may represent a novel mechanism for the effects of PMSCs on placental homeostasis in normal pregnancy. However, miR-30a expression significantly increased in the preeclamptic UCMSCs, which impaired the anti-inflammatory effects on macrophages. Furthermore, LncRNA MALAT1-overexpressed PMSCs promoted M2 macrophage polarization and this effect was mediated by MALAT1-induced IDO expression, however, the expression of MALAT1 was significantly reduced in both umbilical cord tissues and PMSCs in patients with severe PE (Li et al., 2017). Thus, preeclamptic PMSCs may participate in the immune dysregulation of the maternal-fetal interface.

##### 4.2.4.3 Interaction Between PMSCs and T Cells

T cells account for 10%–20% of the leukocyte population in human decidua during the first trimester of pregnancy. In human decidua T lymphocytes, 45%–75% of these cells are CD8^+^ cells and 30%–45% of these cells are comprised of CD4^+^ cells. Thereinto, approximately 50% of the CD4^+^ cell population possess the activation and memory CD25^dim^ phenotype, 5%–30% are T-helper (Th) 1 cells, 5% are Th2 cells, and 2% are Th17 cells; A significant enrichment (5%) in CD25^bright^Foxp3^+^ regulatory T (Treg) cells and a more homogenous suppressive phenotype was observed (Mjösberg et al., 2010). Though moderately abundant in the human decidua, T cells may play a pivotal role in immune regulation at the fetal-maternal interface via being skewed from Th1-like toward Th2-like immunity. Besides, Treg cells are likely to suppress fetus-specific and nonspecific responses when the maternal immune cells come into contact with fetal antigens, exerting a prominent protective role (Erlebacher, 2013; La Rocca et al., 2014). However, the abnormal alteration in the recruitment, expansion, and phenotype of T cells has been observed in the pregnancy complications such as PE (Sasaki et al., 2007).

First, PMSCs and their CM strongly suppress T lymphocyte proliferation *in vitro* (Wolbank et al., 2007; Magatti et al., 2008; Roelen et al., 2009; Kronsteiner et al., 2011; Kang et al., 2012; Rossi et al., 2012; Manochantr et al., 2013; Liu et al., 2014; Yamahara et al., 2014), thereinto, fetal PMSCs demonstrated a significantly higher inhibitory capacity, compared with maternal PMSCs, which may be consistent with a protective mechanism adopted by fetal MSCs towards the mother’s immune activity (Roelen et al., 2009). Second, PMSCs and their CM are also able to effectively inhibit Th1 and Th17 differentiation rather than Th2 differentiation (Liu et al., 2014; Pianta et al., 2015), which may be beneficial to the immunoregulation at the fetal-maternal interface. Third, PMSCs can also modulate the immune responses via *de novo* induction and expansion of Tregs. AMMSCs and their CM promote the proliferation of Treg cells and stimulate the secretion of immunoregulatory factors such as IL-10 (Pianta et al., 2015). However, in PE patients, the expression of miR-181a was increased in the UCMSCs and DBMSCs, which disturbs local immune balance by inhibiting T cell proliferation by blocking the activation of the TGF-β signaling pathway and impairing immunosuppressive properties (Liu et al., 2012). Hence, PMSCs and their secreted factors suppress the inflammatory cytokine production and modulate the differentiation skewness of T cells, while they stimulate the generation of Treg cells in normal pregnancy. On the contrary, the preeclamptic PMSCs are likely to disturb the immune balance of T cells at the mater-fetal interface, thus suppressing early placental development.

## 5 Clinical Trials and PE Therapies Associated With PMSCs

Nowadays, the growing rate and encouraging results of clinical trials (https://clinicaltrials.gov) using PMSCs for multiple diseases (Table 1) indicate the favor of PMSCs in clinical therapeutic strategies (Prather et al., 2009). Nevertheless, up till now, the treatment of PMSCs or PMSCs’ exosome for PE is still in the stage of laboratory experiments. Placental hypoxia is a central pathophysiological change in PE, contributing to trophoblastic dysfunction. With the activation of the JAK2/STAT3 signaling pathway, CMMSCs-mediated autophagy ameliorates the proliferation and invasiveness of trophoblasts under hypoxia (Chu et al., 2021). AFMSCs exosome-mediated autophagy also promotes trophoblastic proliferation under hypoxia through EZH2/mTOR signaling (Chu et al., 2020). Analogously, excessive activation of inflammation and oxidative stress at the uteroplacental interface in PE leads to placental dysfunction (Zhang et al., 2021a; Zhang et al., 2021b). DBMSCs-secreted extracellular vesicles reduced production of pro-inflammatory cytokine IL-6 and levels of lipid peroxidation, thus reducing oxidative stress in ECs induced by lipopolysaccharide or preeclamptic serum *in vitro* (Zheng et al., 2020). Reportedly, exosomal miR-146a-5p and miR-548e-5p from AFMSCs could ameliorate the inflammatory response, thus modulating the proliferation, apoptosis, migration, and oxidative stress in trophoblasts (Yang et al., 2019). Additionally, the conditioned medium of PMSCs has protective effects on lipopolysaccharide-induced pregnant mice via decreasing maternal systolic blood pressure, proteinuria, sFlt-1, IL-6, and TNF-α levels (Nuzzo et al., 2022). Both PMSCs transplantation and PMSCs exosome therapy could ameliorate placental vascular dysplasia, increase placental perfusion, alleviate PE symptoms, and improve pregnancy outcome by regulating the balance of the angiogenic (VEGF) and anti-angiogenic factors (sFlt-1) or directly participating in the repair of placental vessels in a PE-like rat model constructed by N-nitro-l-arginine methyl ester (l-NAME) (Xiong et al., 2018; Huang et al., 2021b; Liu et al., 2021). From this, it seems that PMSCs may be a reasonable alternative approach to prevent and treat PE.

**TABLE 1:** PMSCs as an alternative tool for clinical therapeutic strategies.

	Study Title	Condition	References
1	Study of hCT-MSC in Newborn Infants With Moderate or Severe HIE	Moderate to Severe Hypoxic-ischemic Encephalopathy (HIE); human umbilical cord tissue-derived mesenchymal stromal cells (hCT-MSC)	NCT03635450; https://clinicaltrials.gov
2	Safety Study of PLX-PAD Cells to Treat Pulmonary Arterial Hypertension (PAH)	Pulmonary Arterial Hypertension; placenta-derived adherent cells (PLX-PAD)	NCT01795950; https://clinicaltrials.gov
3	Safety of Intramuscular Injection of Allogeneic PLX-PAD Cells for the Treatment of Critical Limb Ischemia	Peripheral Artery Disease; Peripheral Vascular Disease; Critical Limb Ischemia; placenta-derived adherent cells (PLX-PAD)	NCT00919958; https://clinicaltrials.gov
4	Safety of Intramuscular Injections (IM) of Allogeneic PLX-PAD Cells for the Treatment of Critical Limb Ischemia (CLI)	Peripheral Artery Disease; Peripheral Vascular Disease; Critical Limb Ischemia; placenta-derived adherent cells (PLX-PAD)	NCT00951210; https://clinicaltrials.gov
5	Human Placental Mesenchymal Stem Cells Treatment on Diabetic Foot Ulcer	Diabetic Foot Ulcer; Placental Mesenchymal Stem Cells	NCT04464213; https://clinicaltrials.gov
6	Safety and Efficacy Study of Umbilical Cord/Placenta-Derived Mesenchymal Stem Cells to Treat Type 2 Diabetes	Type 2 Diabetes; Umbilical Cord/Placenta-Derived Mesenchymal Stem Cells	NCT01413035; https://clinicaltrials.gov
7	Safety and Efficacy Study of Umbilical Cord/Placenta-Derived Mesenchymal Stem Cells to Treat Ankylosing Spondylitis (AS)	Ankylosing Spondylitis; Umbilical Cord/Placenta-Derived Mesenchymal Stem Cells	NCT01420432; https://clinicaltrials.gov
8	Safety and Efficacy Study of Umbilical Cord/Placenta-Derived Mesenchymal Stem Cells to Treat Myelodysplastic Syndromes	Myelodysplastic Syndromes; Umbilical Cord/Placenta-Derived Mesenchymal Stem Cells	NCT01129739; https://clinicaltrials.gov
9	A Study to Evaluate the Potential Role of Mesenchymal Stem Cells in the Treatment of Idiopathic Pulmonary Fibrosis	Idiopathic Pulmonary Fibrosis; Placental Mesenchymal Stem Cells	NCT01385644; https://clinicaltrials.gov
10	Evaluate the Safety and Feasibility of Injecting PMD-MSC Into the Penis to Treat the Symptoms of PD	Peyronie’s Disease (PD); Placental Matrix-Derived Mesenchymal Stem Cells (PMD-MSCs)	NCT02395029; https://clinicaltrials.gov
11	Evaluate the Safety and Feasibility of Injecting PMD-MSC Into the Penis to Treat the Symptoms of Mild to Moderate ED	Erectile Dysfunction (ED); Placental Matrix-Derived Mesenchymal Stem Cells (PMD-MSCs)	NCT02398370; https://clinicaltrials.gov
12	Cellular Therapy for In Utero Repair of Myelomeningocele - The CuRe Trial	Myelomeningocele; Placental Mesenchymal Stem Cells	NCT04652908; https://clinicaltrials.gov
13	Allogeneic Mesenchymal Stem Cell for Graft-Versus-Host Disease Treatment	Graft-Versus-Host Disease; BMMSCs; PMSCs	NCT00749164; https://clinicaltrials.gov
14	Intra-articular Injection of MSCs in Treatment of Knee Osteoarthritis	Osteoarthritis; Placenta Derived Mesenchymal Stem Cell	NCT03028428; https://clinicaltrials.gov
15	Efficacy of Bone-marrow-derived and Placenta-derived Multipotent Mesenchymal Stem/Stromal Cells for Osteoarthritis	Osteoarthritis; Placenta-derived Multipotent Mesenchymal Stem/Stromal Cells	NCT04453111; https://clinicaltrials.gov
16	Effect of Implanting Allogenic Cytokines Derived From Human Amniotic Membrane (HAM) and Mesenchymal Stem Cells Derived From Human Umbilical Cord Wharton’s Jelly (HUMCWJ) on Pain and Functioning of Knee Osteoarthritis	Knee Osteoarthritis; Knee Pain Chronic; Joint Disease; Human Amniotic Membrane (HAM); Mesenchymal Stem Cells Derived From Human Umbilical Cord Wharton’s Jelly (HUMCWJ)	NCT03337243 https://clinicaltrials.gov
17	Open-label Multicenter Study to Evaluate the Efficacy of PLX-PAD for the Treatment of COVID-19	COVID-19; ARDS; placenta-derived adherent cells (PLX-PAD)	NCT04614025; https://clinicaltrials.gov
18	Treatment of Coronavirus COVID-19 Pneumonia (Pathogen SARS-CoV-2) With Cryopreserved Allogeneic PMMSCs and UC-MMSCs	COVID-19 Pneumonia; Placenta-Derived Multipotent Mesenchymal Stromal Cells	NCT04461925; https://clinicaltrials.gov

## 6 Conclusion

**FIGURE 1:**
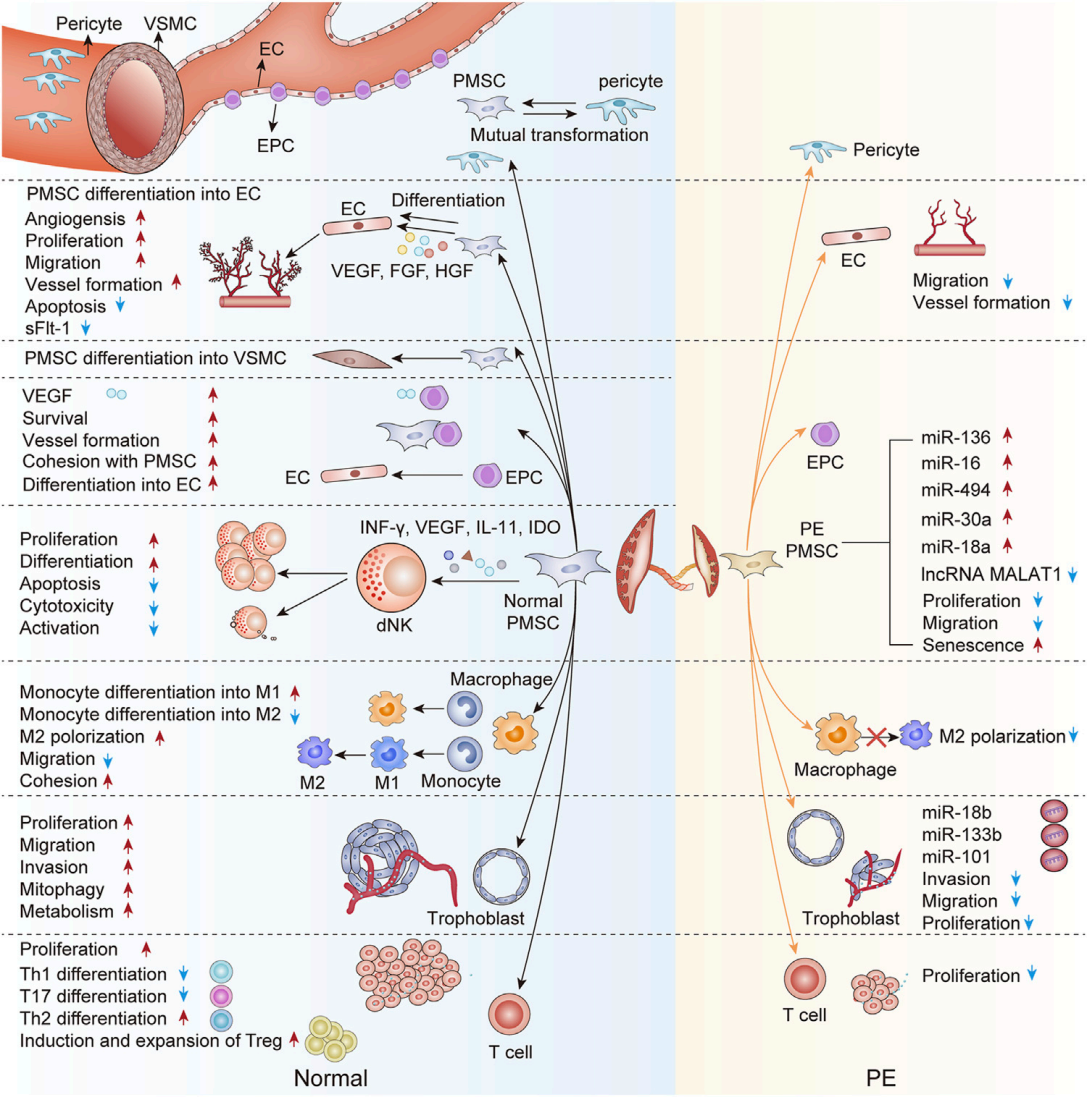
An interactive network of PMSCs and other component cells in the normal and preeclamptic placenta. This figure proposes unique crosstalk between PMSCs and other component cells at the maternal-fetal interface. Briefly, PMSCs may differentiate into the constitutive cells of vessel walls (ECs, VSMCs, and pericyte) in the early placenta vasculogenesis. Besides, owing to the paracrine action, PMSCs in the normal pregnancy augment the placental angiogenesis, enhance the trophoblastic function, and modulate the placental immune status. On the contrary, the preeclamptic PMSCs are characterized by the detrimental interactions with other component cells in the placenta, the underlying mechanism of which may partly be the abnormal PMSCs paracrine in PE. EPC, endothelial progenitor cell; EC, endothelial cell; VSMC, vascular smooth muscle cell; dNK, decidual NK cell; M1, M1 macrophage; M2, M2 macrophage; Th1, T-helper 1 cell; Th2, T-helper 2 cell; Th17, T-helper 17 cell; Treg, regulatory T cell; sFlt-1, soluble fms-like tyrosine kinase-1; VEGF, vascular endothelial growth factor; bFGF, basic fibroblast growth factor; HGF, hepatocyte growth factor; sFlt-1, soluble fms-like tyrosine kinase-1; ANG, angiotensin; IL-11, interleukin-11; IDO, indoleamine2,3-dioxygenase; miRNA, micro-RNA; LncRNA, long non-coding RNA; PE, preeclampsia.

Recently, single-cell RNA sequencing (scRNA-seq) has been performed on sorted placental cells from first-trimester human placentas. Thereinto, new subtypes of the PMSCs were identified from the first-trimester villous mesenchymal core and cell-type-specific genes were defined such as Delta-like homolog 1 (DLK1) (Liu et al., 2018). Based on GO analysis of the differentially expressed genes (DEGs) between the two subtypes, it appears from the enriched terms that PMSCs with high DLK1 expression participate in the vascular formation and mesenchyme development, whereas PMSCs with low DLK1 expression indicates involvement in the regulation of cell migration and adhesion. These advances in detective methods reveal the regulatory relationships between PMSCs and other placental cells and uncover the real trajectories of PMSCs in the development of the placenta. Concretely, PMSCs may initiate early placental vasculogenesis *via* differentiating into constitutive cells of vessel walls. Additionally, with the active paracrine, PMSCs augment the placental angiogenesis, enhance the uterine spiral artery remolding, and modulate immune status at the uteroplacental interface. However, the detrimental alterations in the biofunction and paracrine of the preeclamptic PMSCs disturb the cellular crosstalk with other component cells in the placenta, thus participating in the development and severity of PE (Figure 1). Furthermore, PMSCs transplantation or PMSCs’ exosome therapies tend to improve the placental vascular network and trophoblastic functions in the PE model, suggesting PMSCs may be a novel and putative therapeutic strategy for PE.

The field of PMSCs study thus far has been thin on the ground to date and is far from sufficient. Future studies are needed to delineate how PMSCs differentiating participated into the vasculogenesis in the early placenta development. Besides, spatial transcriptomics may be recommended to discover the fate more clearly on PMSCs subsets as well as explore the spatiotemporal specificity. Comparative research on the normal and preeclamptic PMSCs is also necessary, particularly the precise analysis of their paracrine constituent. Furthermore, more clues concerning abnormal interaction networks between preeclamptic and other placental cells need to be discovered. Comprehensive therapies targeting PMSCs and cellular interaction networks may also provide new insights into the prevention and treatment of PE. In addition to the ethical issue, more research is needed to fully evaluate the effectiveness and fetal-maternal safety of PMSCs treatment, thus providing sufficient theoretical basis for clinical trials in the future.
